# Toward universal representations of the living: Physiological invariance for transportable medical AI

**DOI:** 10.1371/journal.pdig.0001610

**Published:** 2026-07-28

**Authors:** Alexandre Vallée

**Affiliations:** Department of Epidemiology and Public Health, Foch Hospital, Suresnes, France; The University of British Columbia, CANADA

## Abstract

Medical artificial intelligence (AI) models often degrade when deployed beyond their training environment, suggesting reliance on context-specific correlations rather than stable physiological structure. This article considers whether improved transportability may require representation learning strategies aligned with biological mechanisms expected to persist across populations, devices, and care pathways. Physiological invariance is introduced as the hypothesis that outcome-relevant predictive relationships may be mediated by latent physiological processes that are more stable across environments than observed measurements shaped by workflows or data acquisition. Multimodal self-supervised learning combined with mechanism-informed regularization may help identify such environment-stable structure, although empirical validation remains limited. Physiological invariance is not proposed as a sufficient or necessary condition for generalization, but as a candidate structural explanation for transportability in domains where shared biological mechanisms exist.

## Introduction

Most medical AI systems excel where they are developed and degrade when deployed across hospitals, populations, or time [1]. Recent empirical efforts in foundation models for healthcare further highlight both the promise and fragility of large-scale pretrained systems when deployed across institutions [2–4].

This instability suggests reliance on context-specific correlations rather than outcome-relevant biological structure expected to persist across environments. Improved transportability may therefore depend on learning representations aligned with physiological invariants, environment-stable mechanisms inferred from multimodal observations, rather than proxy patterns induced by data collection or care processes. Concrete clinical examples help clarify this concept. In cardiovascular medicine, the invariant is not a fixed blood pressure value, but the structured relationship linking arterial pressure, pulse pressure, flow, vascular resistance, and arterial compliance, classically formalized by Windkessel-type models and measurable through arterial waveforms, cuff blood pressure, photoplethysmography, and echocardiography [5,6]. In endocrine regulation, the invariant is not an absolute hormone concentration, but the feedback architecture and temporal coupling among hypothalamic, pituitary, and peripheral hormones, such as GnRH, LH, FSH, estradiol, progesterone, TSH, and free thyroxine trajectories [7,8]. In laboratory medicine, routine biomarkers also encode constrained physiological relationships, including hemoglobin–hematocrit–red blood cell count consistency and creatinine dynamics constrained by glomerular filtration physiology [9,10]. These examples illustrate that physiological invariants are operationally measurable relationships among biomarkers, signals, and imaging-derived features, rather than abstract universal constants.

Physiological invariance is introduced here as a candidate structural explanation for cross-environment generalization. The following sections examine how such structure may be approximated using multimodal self-supervised learning, mechanism-informed constraints, and causality-oriented objectives, and how it may be evaluated through cross-environment calibration, subgroup reliability, and uncertainty monitoring. Physiological invariance is not proposed as a sufficient or necessary condition for generalization, but as a testable hypothesis in domains where shared biological mechanisms exist. Generalization in medical AI is thus reframed as a scientific problem concerning the stability of outcome-relevant mechanisms across environments. The framework presented here is conceptual and programmatic; empirical validation of physiological invariance as a design principle remains an open research challenge.

### Terminological clarification

For clarity, we define three key terms used throughout this article.

**Invariant representations** refer to latent representations whose predictive relationship to clinical outcomes remains stable across predefined environments (e.g., hospitals, time periods, acquisition protocols), reflecting transportable generative mechanisms rather than environment-specific correlations [11,12].

**Mechanism-informed learning** denotes the incorporation of biological or physiological knowledge, such as conservation constraints, dynamical systems structure, or known feedback loops, into model training as soft regularization terms rather than deterministic rules [13,14].

**Shortcut learning** describes the phenomenon whereby models rely on spurious but predictive features that are statistically associated with outcomes in the training data yet not causally or physiologically related to the underlying condition, leading to failure under distribution shift [15,16].

These concepts are related but not interchangeable. Invariant representation denotes the representational objective: a latent structure whose relationship to the clinical outcome remains stable across predefined environments. Mechanism-informed learning denotes a training strategy: the use of biological or physiological priors as soft constraints to encourage such representations. Shortcut learning denotes a failure mode: reliance on environment-specific cues that are predictive in the development setting but not causally or physiologically related to the target condition.

These terms are used consistently throughout the manuscript to distinguish stable biological structure from context-dependent statistical artifacts.

## 1. Why medical AI fails to generalize across sites

Despite impressive internal validation, model performance often degrades when a system is moved from one hospital to another or deployed months after development. In medical imaging, for example, seemingly state-of-the-art models trained to detect pneumonia in chest radiographs have shown marked performance drops and shortcut behavior when evaluated out-of-site, revealing reliance on non-physiological cues such as device-brand artifacts or site-specific protocols. Such failures are not anecdotal but symptomatic of learning pipelines that optimize predictive accuracy within a single environment while ignoring the causal instability of features tied to workflows, coding practices, or local prevalence. In parallel, widely used risk algorithms can encode structural inequities when they learn on proxies, such as cost of care, that entangle biology with access and utilization; at a given risk score, marginalized patients may be systematically sicker, with major implications for equitable allocation of care. These observations motivate an explicit shift from pattern fitting to mechanism-seeking representations [17,18].

Consider a hospital early-warning system trained to predict acute deterioration from routine vital signs and laboratory data. In the development hospital, the model achieves excellent discrimination by exploiting subtle correlations between laboratory ordering patterns, monitoring frequency, and local care workflows. When deployed in a second hospital, however, performance degrades sharply, not because human physiology has changed, but because laboratory timing, clinical thresholds, and escalation protocols differ [19–21]. A representation anchored in physiological invariants, such as coherent cardio-respiratory dynamics or mass-balance constraints across biomarkers, would be less sensitive to these contextual shifts. In such a setting, physiological invariance would be operationalized through measurable coupled trajectories rather than isolated predictors. For example, impending circulatory or respiratory decompensation should be reflected in coherent changes across heart rate, blood pressure or pulse pressure, respiratory rate, oxygen saturation, lactate, renal function markers, and possibly arterial blood gas variables [22]. These variables are not interchangeable features: they are linked through cardio-respiratory coupling, tissue perfusion, oxygen delivery, acid–base regulation, and renal clearance. By contrast, laboratory ordering frequency, monitoring intensity, device type, or ward-specific escalation rules may be highly predictive within one hospital but are not physiological invariants. A model that generalizes should therefore rely more on the coherence of these biological trajectories than on local care-process proxies.

This example illustrates how generalization failure often reflects reliance on environment-specific correlations rather than instability of the underlying biology, motivating invariance as a design principle rather than a post hoc fix. A systematic review of deep learning algorithms for radiologic diagnosis found that most models experienced measurable performance degradation on external validation, with AUROC decreases commonly ranging from 0.05 to 0.15 depending on task and case-mix differences [19]. In intensive care prediction models, external validation studies have reported average AUROC reductions on the order of 0.04–0.10, though variability across tasks and populations remains substantial [20,21]. Similarly, evaluations of predictive risk scores in intensive care settings have documented average decreases in AUROC during external validation, underscoring the persistent challenge of clinical model generalizability [20].

## 2. Empirical exemplars: Why generalization fails (and what seems to help)

Empirically, “generalization failure” is not a single phenomenon and can reflect distinct mechanisms. In medical imaging, cross-site performance drops have been repeatedly documented for pneumonia detection on chest radiographs, with analyses showing sensitivity to non-pathological cues and site- or device-specific signatures consistent with shortcut learning [22]. However, some degradation is also plausibly explained by differences in case mix and prevalence (label shift), which can alter calibration and apparent discrimination even when underlying feature–outcome relationships are stable; recent work has proposed prevalence-aware adjustment workflows that can partially mitigate such shifts without additional labels [23]. In addition, label instability and heterogeneous labeling schemas represent a separate source of “conceptual shift,” particularly in imaging where ground-truth definitions and annotation processes vary across institutions; reviews have highlighted labeling noise and schema variation as underappreciated contributors to brittleness in deployed imaging AI [24]. These three drivers, shortcut learning, prevalence/case-mix shift, and label instability, are not mutually exclusive, which motivates explicit environment documentation and shift-aware evaluation rather than attributing failures to a single cause [25].

Evidence for mechanism-informed learning improving transportability is currently strongest in domains where physiology is well-characterized and measurements are high frequency (e.g., waveforms). For example, physics-informed neural network models have been applied to physiological time series to extract cardiovascular information with limited ground-truth supervision, illustrating how mechanistic constraints can reduce reliance on purely correlational cues in settings with identifiable dynamics [22,26]. More broadly, mechanistic ML/data-assimilation perspectives argue that embedding scientific structure can improve robustness when the mechanistic component is sufficiently accurate and the data support identifiability [27]. At the same time, direct comparative evidence that SSL combined with mechanistic regularization systematically outperforms simpler domain adaptation strategies across healthcare tasks remains limited; this Perspective therefore frames SSL + mechanistic regularization as a promising research direction whose benefits must be demonstrated empirically against strong baselines (e.g., output recalibration for prevalence shift, or standard domain adaptation for acquisition shift) [23,28]. These examples also suggest where physiological invariance is most empirically tractable. High-frequency cardiovascular waveforms provide one candidate domain, because pressure, flow, pulse morphology, and vascular compliance are dynamically coupled and can be measured repeatedly across devices and care settings [5,22]. Longitudinal endocrine data provide another example: absolute hormone levels vary across individuals and life stages, but feedback-regulated temporal patterns, pulsatility, and response to perturbation can be modeled as constrained trajectories [7,8]. Routine laboratory panels provide a third, lower-dimensional example, where biomarker constellations such as hemoglobin–hematocrit–red blood cell count or creatinine–eGFR relationships can be used to detect implausible representations or measurement-driven artifacts [9,10]. These domains are useful test beds because the expected physiological relationships are partially known, clinically interpretable, and measurable across environments.

## 3. What invariance should mean in medicine (and what it should not)

“Invariance” is often treated as a modeling trick, data augmentation, adversarial training, or site reweighting, to make models less sensitive to nuisance variation. In clinical science, invariance has a sharper meaning: a property of the data-generating mechanisms that transports under interventions, environments, and sampling shifts.

In medicine, invariance should be understood as structured transportability rather than numerical constancy. The relevant question is not whether biomarker values, imaging features, or physiological signals are identical across individuals, but whether the relationships linking these measurements to latent biological states remain sufficiently stable across environments to support prediction. This interpretation distinguishes physiological invariance from generic domain-invariant feature learning: the objective is not merely to remove site information, but to preserve biologically meaningful structure while reducing reliance on workflow-induced or acquisition-specific artifacts [11,12]. Physiological invariance does not imply the absence of variability.

Physiological invariance does not imply uniformity across individuals. Rather, it refers to the stability of generative mechanisms while allowing parameters, boundary conditions, and responses to vary across persons. In cardiovascular physiology, for example, the coupling between flow, resistance, and pressure remains governed by conservation laws, yet arterial compliance, vascular tone, and autonomic balance differ substantially by age, sex, and comorbidity [29]. Similarly, endocrine feedback systems follow conserved regulatory architectures, while hormone amplitudes, pulse frequency, and sensitivity to perturbation vary across individuals and life stages [7].

This distinction parallels hierarchical and multi-level modeling in statistics, where structural equations are shared while parameters are partially pooled across groups. In biological systems, invariance lies in the form of interactions rather than in fixed numeric values. Gene–environment interactions further illustrate this principle: regulatory pathways may be conserved, yet their expression depends on genotype, developmental exposure, and environmental context [30].

For medical AI, the implication is that invariant representations should encode stable mechanistic relationships while preserving axes of biologically meaningful variation. A representation that erases sex-specific pharmacokinetics or age-dependent immune responses would not be physiologically faithful, even if it appears statistically stable. Thus, invariance must be understood as structured transportability, not homogenization. The aspiration of “universal” representations is to locate individuals within a shared physiological coordinate system, not to collapse them into a single prototype.

The concept of physiological invariance is advanced here as a guiding hypothesis rather than a universal solution. Its scope is necessarily bounded. Not all clinical prediction tasks admit invariant representations. When outcome definitions, treatment effects, or healthcare infrastructures differ fundamentally across environments, no shared latent structure may exist that guarantees transportability. In such settings, environment-specific modeling strategies may be more appropriate than attempts to enforce invariance. Moreover, environments are not always nuisance variation. Differences in care pathways, resource availability, or therapeutic interventions can exert genuine causal influence on disease trajectories. Abstracting away such contextual factors in the name of invariance may inadvertently remove clinically meaningful signal. Transportability therefore depends on distinguishing noise from causally relevant context. Physiological knowledge itself is incomplete, evolving, and sometimes contested. Mechanism-informed regularization reflects current scientific understanding and is therefore provisional. As biomedical knowledge advances, the assumptions embedded in invariant representations may require revision. In this sense, invariance is not a static property but a moving target shaped by scientific progress. Universal representation should also not be conflated with universal generalization. The existence, strength, and scale of invariant structure are empirical questions rather than theoretical guarantees. In some domains, only partial transportability may be achievable, and acknowledging these limits is essential to avoid overclaiming. Physiological invariance functions less as a doctrinal assertion and more as a research program: an effort to identify when stable biological structure meaningfully supports cross-environment reliability, and when it does not.

If invariance is understood as stability of underlying biological mechanisms across environments, the next question becomes operational: how can such stable structure be learned from heterogeneous clinical data? The answer is unlikely to lie in single-modality modeling. Because physiology is expressed across signals, images, laboratory trajectories, and text, invariant structure must be inferred from their joint organization. This motivates a shift from purely predictive modeling toward multimodal representation learning guided by mechanism-informed constraints.

These considerations establish the conceptual scope of physiological invariance: it is neither a claim of biological uniformity nor a guarantee of universal generalization. The next section provides a minimal formal characterization of this hypothesis and clarifies its relationship to causal parent variables and latent physiological mechanisms.

## 4. A minimal formal characterization of physiological invariants

**Fig 1:**
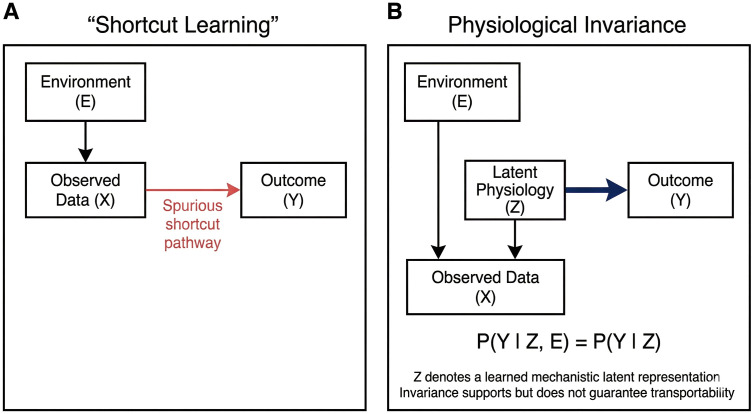
Conceptual schematic of physiological invariance in medical AI. **(A)** Shortcut learning arises when predictive models exploit environment-dependent correlations between observed data *X* and outcomes *Y*. In such cases, environmental factors *E* (e.g., site, acquisition protocol, or clinical workflow) influence measurements and induce spurious associations that degrade performance under distributional shift. **(B)** Physiological invariance aims to recover representations aligned with stable biological processes. A latent physiological representation *Z*, inferred from multimodal observations, generates both observed features *X* and clinical outcomes *Y*, while environmental factors affect measurements without altering the underlying physiological mechanism. Under this assumption, the predictive relationship satisfies. P(Y∣Z,E)=P(Y∣Z), indicating stability of outcome-relevant structure across environments. Here, *Z* denotes a learned mechanistic latent representation rather than directly observed causal parents of the outcome. Such invariance may support transportability across settings but does not in itself guarantee generalization.


The conceptual boundaries defined above can be expressed more formally within a structural framework (Fig 1). Let *E* denote environments (e.g., hospital, time period), *X* observed multimodal features, *Y* a clinical outcome, and *Z* latent physiological variables. A representation ϕ(X) is physiologically invariant if there exists a subset $Z^{*}=\phi (X)$ such that:

$$\begin{array}{c} P\left(Y|Z*,E\right)=P\left(Y|Z*\right)\;\forall E \end{array}$$

and $Z^{*}$ corresponds to variables that are interpretable as components of stable physiological mechanisms rather than environment-specific artifacts, consistent with invariance principles in causal prediction and invariant risk minimization frameworks [11,12].

This definition differs from generic domain-invariant features, which may remove environment-predictive signals without ensuring biological interpretability. It also differs from identifying causal parents of *Y*, as invariants may correspond to mechanistic latent variables not directly observed but governing multiple measurable projections [31]. Physiological invariance therefore occupies an intermediate position: it requires stability of predictive structure across environments while grounding that stability in biological generative processes.

This distinction is important because physiological invariants and causal parent variables are related but not equivalent. In a structural causal model, causal parents of a clinical outcome are variables with direct causal effects on that outcome under a specified graph and intervention set [30]. Invariant causal prediction and invariant risk minimization formalize related ideas by searching for predictors or representations whose relationship with the outcome remains stable across environments [11,12]. However, physiological invariants refer more broadly to stable generative relationships or latent physiological coordinates that may govern several observable measurements across environments, consistent with the broader agenda of causal representation learning [32]. Such invariants may coincide with causal parents when the latent physiological process directly influences the clinical outcome. However, they may also correspond to upstream mechanisms, mediators, conserved constraints, or measurement-generating processes that are not direct parents of the outcome but still improve transportability by stabilizing the mapping between observed data and biological state. For example, arterial compliance may not always be the direct causal parent of a specific endpoint, yet the pressure–flow–resistance relationship it constrains can help identify hemodynamically meaningful representations across devices or sites. Similarly, endocrine feedback structure may stabilize interpretation of hormone trajectories even when the immediate outcome is defined by a downstream clinical event. Thus, causal parent identification asks which variables directly cause the outcome, whereas physiological invariance asks which biologically interpretable relationships remain stable enough across environments to support transportable prediction.

Importantly, physiological invariance is proposed neither as a strictly necessary nor as a sufficient condition for transportability [33]. Some predictive tasks may generalize across environments despite partial violation of invariance if distributional shifts are limited or controlled. Conversely, even perfectly invariant mechanisms may fail to guarantee transportability when measurement error, intervention effects, or policy differences alter the observable mapping between physiology and outcome. Invariance should therefore be understood as a structural hypothesis that can support transportability when present, rather than as a universal prerequisite or guarantee.

## 5. Relationship to existing generalization strategies

The concept of physiological invariance should be distinguished from established strategies for improving model generalization. Domain adaptation methods aim to reduce discrepancies between source and target distributions, often through feature alignment or adversarial training [34,35]. Transfer learning and fine-tuning leverage pretrained representations to adapt to new tasks or domains with limited data [36]. Domain generalization approaches seek representations that remain predictive across multiple training environments without access to the target domain [36], while distributionally robust optimization (DRO) minimizes worst-case risk within predefined uncertainty sets.

These methods have demonstrated empirical benefits in both general machine learning and healthcare settings [37]. However, they are primarily statistical in orientation: stability is defined in terms of distributional similarity or predictive performance across datasets. By contrast, the notion of physiological invariance proposed here shifts the focus from distributional robustness to mechanistic robustness. Rather than asking which features remain predictive across environments, it asks which relationships reflect underlying biological processes that are expected to transport across contexts. In this sense, domain adaptation, domain generalization, and DRO can be understood as operational tools for managing shift, whereas physiological invariance is proposed as a design principle about what representations ought to encode. These perspectives are therefore complementary rather than mutually exclusive (Table 1). Physiological invariance should therefore be understood not as a competing algorithmic technique, but as a normative principle guiding representation learning in biomedical contexts.

**Table 1:** Comparison of generalization strategies in medical AI and the proposed concept of physiological invariance.

Approach	Primary Objective	Definition of Stability	Typical Methods	Strengths	Limitations in Healthcare Context	Relation to Physiological Invariance
**Transfer Learning**	Adapt pretrained representations to new tasks or domains	Performance retention after fine-tuning	Pretraining + fine-tuning; frozen backbone; parameter-efficient tuning	Efficient use of limited labeled data; strong empirical gains	May transfer spurious correlations learned during pretraining; limited guarantees under distribution shift	Can provide initialization, but does not ensure mechanism-level stability
**Domain Adaptation**	Reduce discrepancy between source and target distributions	Alignment of feature distributions across domains	Adversarial feature alignment; discrepancy minimization; reweighting	Effective when target domain data are accessible	Requires access to target data; may align non-biological artifacts; reactive rather than principled	Can correct distribution mismatch but does not define what should be invariant
**Domain Generalization**	Learn predictors stable across multiple training environments	Consistent predictive performance across observed domains	Invariant risk minimization (IRM); meta-learning; environment-conditioned training	No access to target domain required; explicit multi-environment training	Stability defined statistically; assumes environments sufficiently diverse; may miss latent confounders	Closest statistical analogue; lacks explicit biological grounding
**Distributionally Robust Optimization (DRO)**	Optimize worst-case performance under uncertainty sets	Bounded performance degradation under distribution shift	Group DRO; adversarial risk minimization; uncertainty sets	Protects minority or worst-case groups; fairness-aware variants	Robustness limited to predefined shift classes; no causal interpretation	Addresses risk robustness, not mechanistic transportability
**Causal Representation Learning**	Identify latent variables aligned with data-generating mechanisms	Stability of conditional distributions under interventions	Invariant causal prediction; disentanglement; structural modeling	Theoretically grounded in causal inference	Strong assumptions; requires environment diversity; limited empirical validation in healthcare	Conceptually aligned; physiological invariance can be viewed as a domain-specific instantiation
**Physiological Invariance (proposed)**	Encode biologically grounded mechanisms that transport across environments	Stability of generative physiological relationships under perturbation	Multimodal SSL + mechanism-informed constraints + causal objectives	Anchors representations in biological structure; integrates modalities; conceptually aligned with clinical science	Depends on quality of mechanistic knowledge; sensitive to data quality; empirical validation still needed	Proposed design principle complementing statistical robustness methods

IRM, invariant risk minimization; DRO, distributionally robust optimization; SSL, self-supervised learning; EHR, electronic health record; ML, machine learning.

Having distinguished physiological invariance from existing statistical generalization strategies, the next question is methodological: how can representations be learned so that they preferentially encode biological structure rather than environment-specific correlations? This motivates the shift from generalization theory to multimodal and mechanism-informed representation learning.

## 6. Learning physiology: Multimodal and mechanism-informed representations

Every clinical modality, vitals, laboratory panels, echocardiograms, CT, free-text notes, proteomics, wearables, offers a partial projection of the same hidden physiological state. To approach invariance, we first need shared latent representations that integrate these projections. Self-supervised learning (SSL) provides the right inductive bias: by predicting masked signals, aligning modalities, or contrasting views, SSL can discover structure without task labels that themselves carry local biases. In healthcare, SSL has shown early promise on EHR sequences, signals, and images, and it naturally scales across institutions. Yet SSL alone will learn whatever is abundant, even if non-physiological. A promising direction is to guide SSL toward biological structure: constrain the latent geometry and dynamics to respect known mechanisms (e.g., conservation laws, hormonal kinetics, hemodynamic coupling), and penalize solutions that attribute predictive power to site-specific artifacts [38]. Large multimodal pretraining efforts integrating imaging, clinical text, and structured EHR data demonstrate improved transfer performance, yet still exhibit degradation under distribution shift, underscoring the need for mechanism-informed invariant representations [39–42]. Beyond invariant risk minimization, recent work in causal representation learning and disentanglement aims to recover latent factors aligned with underlying generative mechanisms, providing a complementary pathway toward transportable models [32,43,44].

### 6.1. Why self-supervised learning and its limits

Self-supervised learning (SSL) is particularly attractive in healthcare because labeled clinical outcomes are sparse, noisy, and institution-specific, whereas raw multimodal signals are abundant. By training models to predict masked segments, align modalities, or contrast temporal views, SSL can learn structured representations without relying on downstream labels that may encode local practice patterns [38]. Large-scale pretraining has demonstrated that SSL improves sample efficiency and transferability across tasks in medical imaging and EHR data [1,38]. In the context of physiological invariance, SSL provides a mechanism to discover shared latent structure across modalities, an essential step toward capturing stable biological processes.

However, SSL is not inherently mechanism-aware. Without constraints, SSL optimizes for statistical predictability, which may include site-specific artifacts or workflow regularities. Empirical analyses of shortcut learning show that models can internalize non-biological correlations even when trained with large self-supervised objectives [15]. In healthcare data, additional risks include label leakage through temporally proximal features, modality imbalance (e.g., dense imaging vs sparse omics), and non-stationarity due to evolving clinical guidelines or documentation practices [37].

Furthermore, contrastive objectives may encourage representations that separate environments rather than align them, potentially encoding hospital or device signatures unless explicitly regularized. For this reason, SSL should be paired with environment-aware objectives and mechanism-informed constraints to steer representation learning toward biologically plausible latent factors. In this framework, SSL serves as a scalable representation engine, while invariance and mechanistic regularization provide the normative guidance about what should be preserved across contexts.

Mechanism-informed learning extends a long tradition of physiological modeling. Classical examples include the Hodgkin–Huxley equations for membrane excitability [45], Guyton’s systems-level model of circulatory regulation [46], compartmental pharmacokinetic models of distribution and clearance [47], the minimal model of glucose–insulin dynamics [48], and biochemical systems theory or systems biology frameworks describing feedback, control, and network organization [49,50]. These models show that physiological variables become interpretable when embedded in dynamical or compartmental structures. The goal of mechanism-informed AI is therefore not simply to attach biological labels to latent variables, but to regularize learned representations using established physiological relationships, constraints, and systems-level dependencies.

Physics-informed neural networks demonstrate how to embed differential equations and constraints into learning objectives. In biomedicine, the “physics” is broader: it includes physiology, pharmacokinetics/pharmacodynamics, mass balance, tissue mechanics, and homeostatic control. By using weakly supervised or differentiable constraints, e.g., enforcing plausible endocrine trajectories across time, Windkessel-consistent arterial dynamics, or mass-balance across compartments, one can tether latent variables to interpretable biological processes [13]. The goal is not to hard-code the body but to regularize learning so that latent factors become candidates for mechanistic variables rather than cryptic site encodings. Such constraints should be paired with causal-oriented objectives like invariant risk minimization or anchor regression to align representations with environment-stable structure [14]. In physics, invariance principles (Lorentz, gauge, conservation) define the very structure of theories. Medicine lacks such formal invariance laws, yet mechanism-informed constraints, mass balance, hormonal feedback, vascular coupling, play an analogous role. Embedding such constraints into learning systems could provide a principled way to approximate stable biological structure.

If invariant representations are the objective, evaluation must go beyond held-out random splits. First, geographically and temporally external validation should be primary, not optional. Second, calibration, the agreement between predicted and observed risks, must be measured across subgroups and environments. Empirical work has shown that even when discrimination remains relatively stable, calibration slope and intercept may shift substantially across environments, particularly when baseline prevalence differs or documentation practices change [51]. However, the magnitude and clinical relevance of such drift vary by task and deployment setting. Modern deep nets are often miscalibrated, but simple post-hoc methods like temperature scaling can help; nevertheless, calibration that only holds in-domain is insufficient for clinical reliability. Third, distributionally robust criteria should be reported, including worst-group performance and counterfactual fairness analyses to ensure that predictions do not hinge on protected attributes or their proxies. Finally, prospective monitoring is necessary to track drift, recalibrate, and halt models when invariance breaks [51].

### 6.2. Practical constraints and epistemic limits of mechanism-informed learning

The feasibility of embedding mechanistic constraints into clinical machine learning systems depends critically on data resolution and measurement fidelity. Many routinely collected health datasets lack the temporal granularity required to support differential-equation-based constraints in a statistically identifiable manner. Sparse laboratory trajectories, irregular sampling in electronic health records, and snapshot imaging data may be insufficient to reliably constrain dynamical systems models [52,53]. In such settings, strong mechanistic penalties may be mathematically elegant yet empirically underdetermined.

Moreover, physiological knowledge is incomplete and often population-specific. Gene–environment interactions, ancestry-associated variation, and life-course effects can alter physiological responses in ways not captured by canonical models [30,54]. Embedding mechanistic priors without accounting for such structured heterogeneity risks introducing hidden assumptions or reinforcing outdated physiological abstractions.

There is also a documented risk of over-regularization in invariant and mechanism-guided learning frameworks. Theoretical analyses of invariant risk minimization have shown that enforcing invariance under violated assumptions can degrade performance or obscure relevant signal [33]. Similarly, physics-informed neural networks may suffer from bias when governing equations are mis-specified or when observational noise dominates constraint terms [14].

Then, evolving clinical practice, treatment protocols, and measurement technologies imply that the observable mapping between physiology and outcomes is not static. Dataset shift literature in healthcare emphasizes that changes in documentation patterns, treatment availability, or case-mix can meaningfully alter predictive relationships over time [37]. In such contexts, mechanistic constraints must remain adaptable rather than fixed.

Thus, mechanism-informed learning should be implemented as tunable, empirically validated priors applied selectively in domains where sufficient temporal structure and mechanistic maturity exist. Its integration into self-supervised learning pipelines is best understood as conditional and iterative rather than universally actionable.

### 6.3. Soft constraints rather than hard-coded rules

Mechanism-informed constraints should be understood as soft regularizers rather than hard-coded physiological rules. In physics-informed neural networks, governing equations are typically incorporated as penalty terms in the loss function, allowing the model to balance empirical fit and physical consistency rather than enforcing exact compliance [14]. A similar principle applies in biomedical contexts. Physiological knowledge is often incomplete, context-dependent, and population-specific; imposing rigid mechanistic equations risks mis-specification and systematic bias.

Overly strong constraints may suppress meaningful heterogeneity or force representations toward incorrect structural assumptions. For example, enforcing uniform endocrine kinetics or vascular dynamics across all individuals could obscure sex-specific or comorbidity-related variation. In such cases, mechanistic priors may function as a source of bias rather than stability.

To mitigate this risk, mechanism-informed objectives should be implemented as tunable regularization terms with empirically calibrated weights. Cross-environment validation can help determine whether constraints improve transportability without degrading subgroup performance. In this framework, mechanistic knowledge acts as an inductive bias guiding representation geometry, not as a deterministic simulator embedded within the model.

Physiological invariance therefore relies on balancing data-driven flexibility with biologically motivated priors. The goal is not to replace learning with equations, but to prevent learning from converging toward environment-specific shortcuts when biological structure offers a more stable explanatory axis.

### 6.4. Mathematical forms of mechanism-informed constraints

Mechanism-informed constraints can be implemented through several mathematical forms, depending on the maturity of physiological knowledge and the structure of the data. A generic training objective can be written as:


$$\begin{array}{c} L=L_{\frac{\text{pred}}{\text{SSL}}}+\lambda_{\text{inv}}L_{\text{inv}}+\sum_{m=\text{1}}^{M}\lambda_{m}L_{\text{mech}}^{\left(m\right)}, \end{array}$$

where $L_{\text{pred}/\text{SSL}}$ denotes the supervised or self-supervised learning objective, $L_{\text{inv}}$ penalizes instability across predefined environments, and $L_{\text{mech}}^{\left(m\right)}$ denotes mechanism-informed penalties. The weights $\lambda_{m}$ should be treated as tunable hyperparameters selected through cross-environment validation rather than fixed from in-domain performance alone.

For dynamical physiological systems, mechanism-informed constraints can take the form of ordinary differential equation residuals. If z(t) denotes a latent physiological state and u(t) external inputs or treatments, the model may be regularized by:


$$\begin{array}{c} L_{\text{ODE}}=\sum_{i,t}{∥\frac{dz_{i}(t)}{dt}-f_{\psi }\left(z_{i}(t),u_{i}(t),t\right)∥}^{\text{2}}. \end{array}$$

This formulation is suitable for endocrine feedback, pharmacokinetic/pharmacodynamic dynamics, hemodynamic coupling, or longitudinal biomarker trajectories. When the mechanistic component is only partially specified, hybrid or universal differential equation models can combine known physiological terms with learnable neural components, allowing the model to preserve mechanistic structure while estimating poorly characterized processes from data [14,55,56].

For spatial or spatiotemporal physiological processes, such as tissue perfusion, diffusion, electrophysiology, or biomechanical deformation, constraints may instead be expressed as partial differential equation residuals:


$$\begin{array}{c} L_{\text{PDE}}=\int_{\Omega \times T}{∥\frac{\partial s(x,t)}{\partial t}+N_{\psi }\left(s,\nabla s,\nabla^{\text{2}}s,u\right)∥}^{\text{2}}dxdt, \end{array}$$

with additional boundary or initial-condition penalties when these are clinically meaningful. Such PDE-based constraints are most appropriate when imaging, waveform, or spatially resolved physiological data provide sufficient temporal or anatomical resolution [14,56].

Simpler physiological knowledge can be encoded through algebraic, monotonicity, or inequality constraints. For example, mass-balance relationships, hemoglobin–hematocrit–red blood cell count consistency, feasible physiological ranges, or monotonic responses to known interventions can be represented as:


$$\begin{array}{c} L_{\text{alg}}=\sum_{t}∥Ah_{\theta }(x_{t})-b_{t}{∥}^{\text{2}},L_{\text{ineq}}=\sum_{t}\text{max}(\text{0},g(h_{\theta }(x_{t}))-c{)}^{\text{2}}. \end{array}$$

These lower-dimensional constraints may be more realistic than full differential-equation models in routine electronic health record data, where sampling is sparse and measurement noise is substantial.

Mechanism-informed constraints can also be embedded within latent-variable models such as variational autoencoders. In this setting, modality-specific encoders estimate $q_{\phi }(z|x)$, while the decoder reconstructs observed modalities from latent physiological states. A mechanism-informed variational objective can be written as:


$$\begin{array}{c} L_{\text{VAE}}=-E_{q_{\phi }\left(z|x\right)}\left[\text{log}\;p_{\theta }\left(x|z\right)\right]+\beta D_{\text{KL}}\left(q_{\phi }\left(z|x\right)∥p(z)\right)+\lambda_{\text{mech}}L_{\text{mech}}(z)+\lambda_{\text{inv}}L_{\text{inv}}(z,e). \end{array}$$

Here, *z* represents a latent physiological coordinate, *e* denotes the environment, and $L_{\text{mech}}$ can impose ODE, algebraic, monotonicity, or plausibility constraints on the latent trajectory. Latent ODE or neural ODE decoders provide a natural extension when observations are irregularly sampled over time, because the latent state evolves continuously between observed measurements [57–59].

Excessive mechanistic constraint can be quantified empirically by examining the regularization path of the mechanism-informed model. For a sequence of constraint weights $\lambda_{\text{mech}}$, including $\lambda_{\text{mech}}=\text{0}$ as an unconstrained baseline, models should be evaluated jointly on predictive performance, calibration, biological plausibility, and subgroup reliability. Over-constraint is suggested when increasing $\lambda_{\text{mech}}$ reduces the mechanistic violation score but simultaneously worsens external AUROC or AUPRC, increases Brier score or expected calibration error, reduces net benefit, or widens worst-group performance gaps. Formally, if C(λ) denotes a constraint-violation score and $R_{e}(\lambda )$ the prediction risk in environment *e*, an excessive constraint regime may be suspected when C(λ) decreases while ${\text{max}}_{e}R_{e}(\lambda )$, subgroup-specific risk, or calibration error increases relative to both the unconstrained model and simpler robustness baselines.

Performance degradation is most likely under several conditions: when the mechanistic equation is mis-specified; when the constraint is applied outside the physiological domain in which it was derived; when data are too sparse or noisy to identify the constrained dynamics; when treatment protocols or measurement devices alter the observable mapping between physiology and outcome; or when the constraint suppresses genuine biological heterogeneity across sex, age, comorbidity, ancestry, or disease stage [14,33,37]. In practice, constraint strength should therefore be selected using nested cross-environment validation and predefined tolerance margins. For example, a mechanism-informed constraint should not be retained solely because it improves biological plausibility if it produces a clinically meaningful deterioration in external calibration, decision-curve net benefit, or worst-subgroup performance. The useful region is therefore not the strongest constraint, but the Pareto region in which mechanistic plausibility improves without material loss of transportability, calibration, or clinical utility.

### 6.5. A stepwise protocol for evaluating physiological invariance

Physiological invariance can be evaluated through a stepwise empirical protocol rather than treated as a purely conceptual property. Environments should be explicitly defined before model development. These environments may correspond to hospital sites, intensive care units, calendar periods, acquisition devices, departments, countries, or care pathways.

Environment types should be distinguished because they affect transferability through different mechanisms. Device or acquisition-protocol environments mainly alter the measurement process, creating risks of device-specific shortcut learning in imaging, waveform, or laboratory data [18,23,25]. Department or care-pathway environments affect workflow variables such as test-ordering frequency, monitoring intensity, documentation density, and escalation thresholds, which may be predictive locally without reflecting stable physiology [18,37]. Temporal environments capture drift in guidelines, coding, treatment availability, prevalence, and case mix, often degrading calibration even when discrimination appears preserved [37,51]. Country-level environments introduce broader variation in population structure, healthcare access, clinical practice, and resource availability, requiring external validation and subgroup reliability analyses [19–21,37]. Thus, invariance testing should specify whether an environment primarily induces measurement shift, workflow shift, temporal drift, population shift, or genuine biological effect modification.

In intensive care, for example, environments may be defined using hospital identifiers, ICU units, monitoring systems, or pre/post implementation periods. Publicly available resources provide useful test beds: MIMIC-IV offers longitudinal electronic health record data including measurements, treatments, procedures, diagnoses, and clinical notes [60]; eICU-CRD provides a multicenter ICU database suitable for cross-site validation [61]; and MIMIC-CXR links chest radiographs with free-text radiology reports, enabling image–text evaluation under acquisition and reporting variability [62]. Multimodal benchmarks such as MedFuse further illustrate how clinical time-series data and chest radiographs can be jointly evaluated when modalities are not uniformly available [63].

Explicit comparator models should then be trained. At minimum, three model classes should be compared: (i) a standard empirical risk minimization model trained without environment-aware constraints; (ii) a domain adaptation or domain generalization model designed to reduce distributional mismatch; and (iii) a mechanism-informed invariant model trained with soft physiological constraints and environment-indexed objectives. This comparison is necessary because physiological invariance should demonstrate added value over simpler strategies such as recalibration, reweighting, acquisition-shift correction, or standard domain adaptation [23,28,37].

The core validation procedure should rely on held-out cross-environment testing. One or more environments should be excluded during training and used only for external testing. Discrimination should be reported using AUROC and AUPRC within each environment, together with absolute and relative performance drops between source and target environments. Calibration should be assessed using calibration intercept, calibration slope, Brier score, and expected calibration error. Clinical usefulness should be evaluated using decision-curve analysis or net benefit across clinically relevant thresholds [37,51,64].

Representation-level auditing should complement predictive evaluation. After training, the encoder can be frozen and auxiliary classifiers can be trained to predict hospital site, acquisition device, department, or calendar period from the latent representation. High environment decodability suggests residual contextual contamination, whereas low environment decodability combined with preserved outcome performance supports, but does not prove, physiological invariance. This audit is particularly important because shortcut learning may remain invisible when only aggregate AUROC is reported [15,16].

Biological plausibility should also be assessed directly. For cardiovascular applications, this may involve assessing whether latent trajectories preserve coherent relationships among heart rate, blood pressure, pulse pressure, oxygen saturation, lactate, and renal function markers. For endocrine applications, this may involve testing whether inferred trajectories preserve plausible feedback-regulated hormone dynamics. For laboratory medicine, this may involve verifying whether representations preserve known biomarker constellations, such as hemoglobin–hematocrit–red blood cell count consistency or creatinine–eGFR coupling [5–10]. A useful invariant representation should therefore be evaluated not only by predictive performance, but also by whether it maintains biologically interpretable relationships across environments.

Prospective or rolling-window evaluation is also required. Models should be monitored over time using temporal AUROC/AUPRC drift, calibration drift, population stability index, subgroup-specific calibration, and conformal coverage. A representation should be considered operationally invariant only if it maintains acceptable discrimination, calibration, uncertainty behavior, subgroup reliability, and biological plausibility across sites, time periods, acquisition settings, and clinically relevant subgroups [37,65,66].

### 6.6. Handling missing modalities and asynchronous sampling

Real-world multimodal healthcare data are rarely complete or synchronous. Laboratory values may be measured intermittently, vital signs may be recorded at high frequency, imaging may occur only once or during clinical deterioration, and free-text notes may follow local documentation practices. Therefore, invariance evaluation should not require fully paired multimodal observations. Instead, missingness should be treated as both a technical problem and a potentially informative clinical signal.

**Fig 2:**
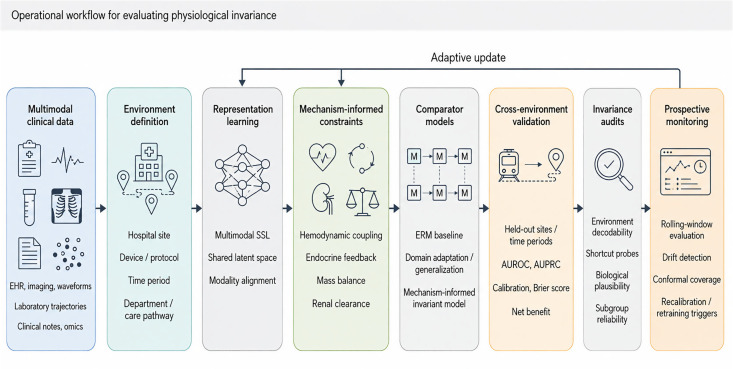
Operational workflow for evaluating physiological invariance in medical AI. The evaluation of physiological invariance begins with multimodal clinical data, including electronic health records, imaging, waveforms, laboratory trajectories, clinical notes, and omics. Environments are explicitly defined using hospital site, acquisition device or protocol, time period, department, or care pathway. Shared representations are then learned through multimodal self-supervised learning and constrained using mechanism-informed priors, including hemodynamic coupling, endocrine feedback, mass-balance relationships, and renal clearance. Candidate models are compared against empirical risk minimization and domain adaptation or domain generalization baselines. Evaluation proceeds through held-out cross-environment validation, representation-level invariance audits, biological plausibility assessment, subgroup reliability analyses, and prospective monitoring. The feedback loop indicates that physiological invariance should be treated as an adaptive lifecycle process, in which model representations and mechanistic constraints are periodically updated according to drift, calibration, uncertainty, and deployment performance.

Several practical strategies can be used (Fig 2). Modality-specific encoders can be trained with shared latent alignment, allowing each modality to contribute when available while avoiding exclusion of patients with incomplete data. This is particularly relevant for multimodal EHR–imaging settings such as MIMIC-IV/MIMIC-CXR, where clinical time-series data and chest radiographs may not be synchronously available for every patient [60,62,63]. Modality dropout during pretraining can expose the model to realistic missing-modality patterns and reduce dependence on any single data stream. Missingness indicators and time-since-last-measurement variables should be explicitly modeled, because the absence of a laboratory test, waveform, or image may reflect clinical workflow, disease severity, resource availability, or local practice rather than random missingness. Models such as GRU-D illustrate how masking indicators and time intervals can be incorporated directly into recurrent architectures for multivariate clinical time series with missing values [67].

Irregularly sampled trajectories should not necessarily be forced into artificial fixed time grids. Continuous-time approaches, such as ODE-RNNs and latent ordinary differential equation models, can model arbitrary time gaps between observations and are therefore well suited to asynchronous clinical measurements [57]. Time-aware attention models provide another strategy by learning representations from sparse and irregularly sampled physiological time series without requiring uniform sampling intervals [68]. These approaches are relevant to intensive-care datasets such as HiRID, where bedside monitoring may be high frequency while laboratory and treatment variables remain irregularly sampled [69].

Importantly, missingness itself should be audited across environments. If one hospital obtains lactate measurements systematically earlier than another, if chest radiographs are acquired under different escalation protocols, or if clinical notes vary in density across departments, the model may learn workflow rather than physiology. Therefore, invariance testing should include environment-wise missingness maps, modality-availability profiles, time-since-measurement distributions, and sensitivity analyses comparing complete-case, imputed, and missingness-aware models. A mechanism-informed invariant representation should remain robust when modalities are partially missing while preserving biologically coherent relationships among the available measurements.

The practical components of this evaluation protocol are summarized in Table 2, which distinguishes environment definition, comparator modeling, cross-environment validation, representation-level auditing, biological plausibility testing, missing-modality robustness, asynchronous sampling, and prospective monitoring.

**Table 2:** Stepwise empirical protocol for evaluating physiological invariance in multimodal healthcare data.

Evaluation component	Practical implementation	Example datasets/ settings	Metrics or outputs	Interpretation
**Environment definition**	Define environments before training using site, ICU unit, device, calendar period, department, country, care pathway, or population stratum; specify whether the expected shift is measurement-related, workflow-related, temporal, geographical, population-based, or biological	MIMIC-IV, eICU-CRD, HiRID, MIMIC-CXR	Environment labels; provenance metadata; modality-availability maps; environment-wise missingness profiles	Establishes the source and mechanism of distribution shift against which invariance will be tested
**Comparator models**	Train standard ERM, domain adaptation/domain generalization, and mechanism-informed invariant models under the same outcome definition	Cross-site ICU prediction; image–text radiology models; multimodal EHR–CXR models	AUROC, AUPRC, Brier score, calibration slope/intercept, ECE	Tests whether physiological invariance adds value beyond simpler statistical robustness strategies
**Cross-environment validation**	Hold out one or more environments during training and use them only for external testing	Train on one hospital/site/time period; test on another	ΔAUROC, ΔAUPRC, ΔBrier, ΔECE, net benefit	Quantifies transportability under realistic site, time, country, department, or device shifts
**Representation-level shortcut audit**	Freeze the encoder and train auxiliary classifiers to predict site, device, department, or calendar period from latent embeddings	MIMIC-CXR device/site shifts; ICU unit-level differences; multimodal EHR–imaging models	Environment classifier accuracy; mutual information with environment	High environment decodability suggests residual contextual shortcut learning
**Biological plausibility testing**	Test whether latent trajectories preserve known physiological relationships	Hemodynamics, endocrine trajectories, renal function, hematologic biomarkers	Constraint violation score; trajectory plausibility; preservation of biomarker coupling	Determines whether the representation encodes biologically interpretable structure rather than only statistical stability
**Over-constraint audit**	Evaluate a regularization path across increasing mechanistic constraint weights, including an unconstrained baseline and simpler robustness baselines	Mechanism-informed EHR, waveform, imaging, or multimodal models; nested cross-environment validation	Constraint violation score; ΔAUROC; ΔAUPRC; ΔBrier; ΔECE; calibration slope/intercept; net benefit loss; worst-subgroup gap	Detects when mechanistic plausibility improves at the cost of transportability, calibration, clinical utility, or subgroup reliability
**Missing-modality robustness**	Evaluate complete-case, imputed, modality-dropout, and missingness-aware models	EHR–imaging datasets with incomplete pairing; ICU datasets with irregular labs and vitals	Performance under simulated and observed missingness; modality-specific degradation	Tests whether the model remains robust when modalities are partially unavailable
**Asynchronous sampling robustness**	Model irregular time gaps using time-aware attention, decay-based RNNs, ODE-RNNs, or latent ODEs	HiRID, MIMIC-IV ICU time-series	Error across sampling densities; time-since-measurement sensitivity	Assesses whether temporal irregularity induces workflow-based shortcuts
**Prospective monitoring**	Apply rolling-window evaluation after deployment or temporal split validation	Pre/post implementation periods; quarterly deployment windows	Temporal AUROC/AUPRC drift; calibration drift; PSI; conformal coverage	Tests whether invariance persists over time and identifies when recalibration or retraining is needed
**Subgroup reliability**	Repeat evaluation across clinically relevant strata	Sex, age, comorbidity burden, ethnicity, disease severity	ΔAUROC, ΔFNR, ΔECE, subgroup calibration slope	Ensures that transportability is not limited to the aggregate population

**Abbreviations:** AUPRC, area under the precision–recall curve; AUROC, area under the receiver operating characteristic curve; CXR, chest X-ray; ECE, expected calibration error; eGFR, estimated glomerular filtration rate; EHR, electronic health record; ERM, empirical risk minimization; FNR, false negative rate; GRU-D, gated recurrent unit with decay; ICU, intensive care unit; ODE, ordinary differential equation; PSI, population stability index; RNN, recurrent neural network.

The preceding sections define how physiological invariance could be operationalized at the representation-learning level and evaluated using cross-environment, missingness-aware, and temporally robust procedures. The following research agenda shifts from methodological principles to implementation: how such procedures could be embedded into cross-institutional pretraining, model comparison, and deployment pipelines.

## 7. A concrete and testable research agenda

We propose a three-part agenda that can be executed with existing data assets and regulatory-compatible tooling. First, construct a cross-institutional, multimodal pretraining corpus in which sites, time periods, and acquisition protocols are explicitly annotated as “environments.” Train shared encoders with SSL objectives that align modalities while minimizing environment-predictability from the latent space; when the environment can be decoded from latents, the representation remains contaminated with contextual shortcuts. Second, add mechanism-informed regularizers tailored to the dominant physiology in each modality cluster: circulation for imaging and waveforms, endocrine dynamics for longitudinal labs, and immune ecology for omics. Even coarse constraints, e.g., bounds on feasible trajectories or monotonicity under known interventions, can reduce reliance on spurious non-biological cues. Third, fine-tune minimal task-heads for sentinel clinical outcomes (e.g., impending decompensation, treatment response) using environment-indexed objectives from the invariance literature, and verify environment-wise agreement of optimal heads. In principle, these steps aim to encourage the model to allocate representational capacity toward biological signals that are more likely to transfer. From a regulatory perspective, invariance can be operationalized as a criterion for safe generalization: models whose calibration and fairness hold under prospective shift demonstrate a form of clinical reliability akin to device stability testing. Future IVDR and FDA guidelines could incorporate invariance audits as part of model re-certification. In practice, “environments” need not rely on perfectly standardized metadata, which are often incomplete or inconsistent in real-world health data. Coarse and weak labels, such as hospital site, calendar time, acquisition device, or care pathway, can already capture meaningful sources of distribution shift. When explicit labels are unavailable, environments may be inferred automatically using unsupervised or weakly supervised clustering of data distributions, workflow patterns, or representation geometry. Finally, environment definition is as much a governance and data stewardship problem as a technical one, requiring transparent documentation of data provenance, acquisition changes, and deployment context.

## 8. Practical feasibility in real-world healthcare systems

Translating physiological invariance into routine clinical practice requires addressing structural constraints of real-world health data ecosystems.

### 8.1. Availability of multimodal data

Although large academic centers increasingly host imaging archives, longitudinal laboratory panels, structured EHR data, and free-text clinical notes, true multimodal completeness remains uneven across institutions. Empirical surveys of foundation models in healthcare highlight both the promise and fragmentation of multimodal datasets, often concentrated in tertiary centers [1]. Self-supervised learning has demonstrated scalability across EHR sequences, imaging, and signals even when modalities are partially missing [38]. Importantly, multimodal pretraining does not require that all sites share identical modalities; partial-modality objectives and shared latent representations allow integration across heterogeneous data availability.

### 8.2. Labeling and defining environments

In practice, environment annotation does not require finely curated metadata. Prior work on dataset shift in healthcare shows that coarse identifiers such as hospital site, calendar period, or acquisition device are sufficient to reveal substantial distributional instability [19–21,37]. Even when explicit labels are incomplete, unsupervised detection of distribution shift through covariate clustering or residual monitoring has proven effective in identifying hidden environments [37,53]. Thus, environment definition can be operationalized pragmatically using routinely available provenance information.

### 8.3. Computational demands

Multimodal self-supervised pretraining at scale may require significant computational resources. However, recent advances in parameter-efficient fine-tuning, federated learning, and distributed training have reduced the need for centralized data pooling or full retraining of large models [70,71]. Federated learning frameworks in healthcare demonstrate that cross-institutional model training can be achieved while preserving data locality and regulatory compliance [71]. Mechanism-informed regularizers typically introduce only modest additional computational cost relative to backbone model training, as they operate as differentiable constraints rather than full simulation layers.

### 8.4. Governance and documentation

Finally, feasibility depends as much on data governance as on model architecture. Transparent documentation of data provenance, acquisition protocol changes, and deployment context is likely to be important for defining environments and monitoring shift [37]. Invariance, in practice, is less about idealized harmonized datasets than about embedding shift-aware evaluation and monitoring into routine model lifecycle management.

Together, these considerations suggest that physiological invariance can be pursued incrementally within existing hospital infrastructures, provided that multimodal integration, environment annotation, and computational strategies are aligned with real-world constraints.

Once invariant representations have been defined, trained, and evaluated, the remaining challenge is not only technical but also clinical and regulatory. A model may encode more stable physiological structure yet still fail if its probabilities are miscalibrated, if subgroup performance is uneven, or if deployment drift is not monitored. The following section therefore treats calibration, fairness, uncertainty, and regulation as downstream tests of whether physiological invariance remains clinically reliable in practice.

## 9. Implications for evaluation, fairness, and regulation

The preceding sections outline a representation-level proposal centered on physiological invariance and mechanism-informed learning. The following considerations, fairness, calibration, uncertainty quantification, and regulatory monitoring, should be interpreted as downstream implications for evaluation and deployment rather than as integral components of the representation-learning framework itself. This distinction separates the methodological core of the proposal from broader policy-facing or lifecycle considerations.

If physiological invariance is proposed as a design principle, it must be evaluated using concrete, testable criteria grounded in established methodological standards. First, invariance across environments can be assessed by comparing performance across predefined sites, time periods, or acquisition settings, following recommendations from the dataset shift literature in healthcare [37]. Environment-wise AUROC or AUPRC, together with variance of performance across environments, provide a first-order diagnostic of transportability [20]. In addition, agreement of optimal predictors across environments, as formalized in invariant risk minimization and invariant causal prediction frameworks [11,12], offers a principled test of representation stability. At the representation level, environment-predictability probes, training auxiliary classifiers to decode hospital or device from latent embeddings, can detect contamination by contextual shortcuts, consistent with recent analyses of shortcut learning in medical AI [15].

Second, calibration under distribution shift must be explicitly quantified. Expected calibration error (ECE), calibration slope and intercept, and Brier score are standard measures of probabilistic reliability [51,64]. These metrics should be computed separately for each environment and clinically relevant subgroup. Importantly, empirical studies have shown that discrimination may remain stable while calibration degrades under shift, underscoring the need for environment-specific calibration assessment [37].

Third, fairness should be evaluated using group-wise performance gaps and counterfactual analyses. Differences in AUROC, false-negative rate, or calibration error across demographic strata reflect potential inequities [72]. Counterfactual fairness frameworks provide a more stringent test: predictions should remain stable under hypothetical changes in protected attributes that do not alter underlying biology [73]. While such counterfactual analyses require modeling assumptions, they serve as stress tests for mechanism-aligned representations.

Finally, uncertainty quantification under shift should be monitored prospectively. Conformal prediction methods provide distribution-free guarantees and adaptive prediction sets that widen under distributional change, offering a practical signal of invariance breakdown at deployment [65,66]. Together, these procedures translate physiological invariance from a conceptual aspiration into an empirically auditable property (Table 3).

**Table 3:** Operational evaluation framework for physiological invariance in medical AI.

Dimension	What is tested	Metrics	Practical procedure	Failure signal	Key References
**Cross-site transportability**	Stability of predictive performance across hospitals/time	AUROC, AUPRC per environment; ΔAUROC; variance across sites	Train on source; evaluate on ≥2 external sites; report dispersion	Large inter-site variance; performance collapse	[19–21,37]
**Predictor invariance (IRM consistency)**	Agreement of optimal predictors across environments	IRM loss; environment-specific risk comparison	Train environment-indexed heads; test if same parameters minimize risk across environments	Environment-specific optima	[11,12]
**Representation contamination (shortcut detection)**	Whether embeddings encode contextual artifacts	Accuracy of environment classifier trained on latent space	Freeze encoder; train probe to predict site/device; compare to random baseline	High environment decodability	[15]
**Calibration under shift**	Reliability of predicted probabilities	ECE; calibration slope/intercept; Brier score	Compute metrics per site & subgroup; calibration plots	Preserved AUROC but degraded ECE	[37,51,64]
**Temporal robustness**	Drift over time	AUROC drift; calibration drift; population stability index	Rolling-window evaluation; quarterly recalibration test	Gradual calibration deterioration	[37]
**Group fairness (observational)**	Equity across subgroups	ΔAUROC; ΔFNR; ΔECE; equal opportunity gap	Stratified evaluation by sex, age, ethnicity	Systematic subgroup harm	[18,72]
**Counterfactual fairness**	Stability under protected-attribute perturbation	Prediction difference under counterfactual swap	Structural causal model; simulate attribute swap	Prediction change without biological change	[73]
**Uncertainty under shift**	Confidence reliability in deployment	Conformal coverage; prediction set size	Wrap model with conformal predictor; monitor coverage drift	Overconfident errors; undercoverage	[65,66]

AUROC, area under the receiver operating characteristic curve; AUPRC, area under the precision–recall curve; ECE, expected calibration error; FNR, false negative rate; IRM, invariant risk minimization.

Causal fairness frameworks provide a principled lens: a decision is “counterfactually fair” if the prediction would remain unchanged in a world where only a protected attribute differed. While counterfactuals require modeling assumptions and cannot be fully validated, they are a useful discipline for stress-testing invariance: if latent factors are truly physiological, predictions should remain stable under counterfactual swaps that do not alter biology. Embedding counterfactual audits into model development and post-deployment monitoring extends invariance from environments to individuals and supports regulatory expectations of non-discrimination [73].

The relationship between physiological invariance and fairness warrants careful qualification. Learning representations aligned with biological mechanisms may reduce reliance on spurious proxies for protected attributes, such as care utilization patterns or documentation density, but does not in itself guarantee fairness. Counterfactual fairness requires that predictions remain stable under hypothetical changes in protected attributes that do not alter underlying biology [73], whereas physiological invariance concerns stability of outcome-relevant mechanisms across environments.

These objectives partially overlap but are not equivalent. A representation that is invariant to site-specific artifacts may still encode biologically mediated differences associated with sex, age, or ancestry. Such heterogeneity reflects structured variation in physiology rather than algorithmic bias. Treating all group-level predictive differences as inequitable risks collapsing legitimate biological diversity into a fairness violation, raising concerns of biological essentialism if not interpreted cautiously [72,74].

Conversely, removing all group-associated signal may degrade clinical validity. Sex-specific pharmacokinetics, age-dependent immune responses, or ancestry-linked genetic variants may be directly relevant to outcome prediction and treatment response. Fairness audits must therefore distinguish between differences arising from access, infrastructure, or proxy entanglement and those grounded in causal biological pathways.

In practice, fairness involves trade-offs with clinical utility. Constraints imposed to equalize error rates across subgroups may alter sensitivity–specificity balance and impact downstream decision thresholds, particularly in resource-limited settings [72]. Physiological invariance should therefore be understood as a potential tool for reducing certain classes of bias, especially those arising from contextual shortcuts, rather than as a sufficient condition for equitable clinical deployment.

Integrating fairness evaluation with mechanism-aware representation learning requires explicit modeling of both biological heterogeneity and social determinants of health, alongside transparent reporting of subgroup calibration and performance trade-offs.

Success should be defined in terms clinicians and regulators can act upon. We recommend reporting, across multiple external sites and periods, the change in discrimination (AUC/AUPRC), expected calibration error and calibration slope/intercept, decision-relevant net benefit curves, and worst-group performance gaps. Where uncertainty quantification is required, conformal prediction can wrap deterministic predictors to produce finite-sample error guarantees and adaptive prediction sets that expand under shift, offering a transparent signal when invariance may be failing at the point of care. Transportability should be demonstrated not only for aggregate cohorts but also for intersectional subgroups defined by sex, age, and comorbidity patterns, precisely where shortcut features are most tempting and most dangerous [65].

Invariance is not an absolute. Genuine biological heterogeneity, gene–environment interactions, and intervention-induced distribution changes imply that no single representation will perfectly transfer everywhere. Mechanism-informed losses risk over-constraining models if mechanistic knowledge is incomplete or population-specific. Methods such as invariant risk minimization can be fragile when environments are not sufficiently diverse or when hidden confounding violates assumptions. These limits counsel humility and argue for continuous post-market surveillance akin to pharmacovigilance, in which recalibration and model updates are transparent, versioned, and governed.

These evaluation criteria provide the methodological basis for an invariance audit. The regulatory question is how such evidence can be documented within existing lifecycle frameworks for AI-based medical devices, without proposing physiological invariance as a new certification category.

## 10. Operationalizing physiological invariance in regulatory frameworks

Regulatory frameworks for AI-based medical devices already provide instruments through which physiological invariance could be evaluated. In the United States, the FDA framework for Software as a Medical Device (SaMD) requires demonstration of analytical validation, clinical validation, and real-world performance monitoring. The FDA’s Good Machine Learning Practice (GMLP) principles further emphasize data representativeness, performance consistency across subpopulations, and lifecycle monitoring. Within this structure, physiological invariance can be operationalized as evidence of stable performance, calibration, and subgroup reliability across predefined environments.

Concretely, an invariance-oriented regulatory submission could include:

(i) geographically and temporally external validation across multiple institutions, consistent with recommendations from dataset shift literature [19–21,37];
(ii) subgroup-specific calibration reporting, including expected calibration error and calibration slope [51,64];
(iii) documentation of environment-aware training objectives and mechanism-informed regularization;
(iv) evaluation of worst-group performance gaps and counterfactual fairness audits [72,73];
(v) prospective drift detection and post-market performance monitoring aligned with SaMD lifecycle expectations.

In this context, an “invariance audit” should not be interpreted as a novel regulatory category, but as a structured extension of existing lifecycle monitoring practices:

At a first level, routine calibration monitoring, including calibration slope, expected calibration error, and subgroup reliability across environments, is already recognized within SaMD performance monitoring frameworks. These procedures assess probabilistic reliability but do not guarantee robustness to distributional or mechanistic shift.

A second level concerns cross-environment stability, evaluated through dispersion of predictive performance across sites, time periods, or acquisition contexts. Environment-wise AUROC or calibration variance provides an operational measure of transportability aligned with existing expectations for external validation and post-market surveillance [37,51,65,73].

A third, more exploratory level involves assessing whether latent representations encode environment-independent structure, for example, by testing environment decodability from learned embeddings or evaluating counterfactual sensitivity under plausible clinical perturbations [72]. These analyses should be viewed as research-oriented evidence of representation stability rather than as currently standardized regulatory requirements.

Framed in this way, invariance audits extend established generalizability and lifecycle monitoring efforts without presuming a regulatory vacuum, translating conceptual transportability into measurable performance stability under deployment-relevant variation.

Under the European IVDR (Regulation EU 2017/746), performance evaluation requires demonstration of scientific validity, analytical performance, and clinical performance, accompanied by continuous post-market performance follow-up. Physiological invariance maps naturally onto these categories: scientific validity corresponds to biological plausibility of the learned structure; analytical performance includes cross-environment stability; and clinical performance encompasses transportability and equitable subgroup reliability.

Importantly, invariance should not be interpreted as a binary certification attribute. Regulatory science increasingly recognizes that AI systems require ongoing surveillance rather than static approval [37,75]. In this light, invariance audits may function analogously to device stability testing or pharmacovigilance: structured, repeatable assessments of calibration, subgroup equity, and drift under real-world deployment conditions. Physiological invariance thus offers a principled lens for interpreting existing regulatory requirements rather than proposing an entirely new regulatory doctrine.

## 11. Concrete equity risks and implementation pitfalls

While physiological invariance may reduce reliance on spurious contextual features, it does not automatically guarantee fairness. Several concrete pitfalls deserve attention.

### 11.1. Proxy entanglement

Predictive models frequently rely on variables that correlate with access to care rather than underlying biology. For example, frequency of laboratory testing, documentation density in clinical notes, or prior healthcare expenditure can act as powerful predictors of adverse outcomes. However, such signals may reflect differences in healthcare access rather than physiological severity, as demonstrated in widely cited bias analyses of population health algorithms [18]. Even multimodal models may encode these proxies unless explicitly audited.

### 11.2. Calibration drift across subgroups

A model may maintain overall discrimination while becoming miscalibrated for specific demographic groups. Empirical and theoretical work has shown that predictive parity and calibration may conflict across groups, particularly when base rates differ [74,76]. In clinical settings, subgroup-specific miscalibration can lead to systematic under-treatment or over-triage despite acceptable global AUROC.

### 11.3. Device and infrastructure bias

Imaging and waveform data are influenced by acquisition devices, sensor placement, and institutional protocols. Prior work has demonstrated that deep learning models can exploit hospital-specific artifacts in radiographs rather than pathology [15,17]. If device type or care pathway correlates with demographic structure, such shortcut learning can reproduce structural disparities.

### 11.4. Mechanism-informed over-constraint

While embedding mechanism-informed constraints may reduce shortcut learning, incomplete or population-specific mechanistic assumptions can obscure meaningful heterogeneity. For example, endocrine trajectories or inflammatory responses may differ systematically by sex, ethnicity, or comorbidity burden. An overly rigid invariance constraint risks enforcing uniformity where biological diversity exists.

### 11.5. Intersectional invisibility

Fairness audits that examine only single attributes (e.g., sex or age independently) may fail to detect compounded disparities in intersectional subgroups (e.g., elderly women with multimorbidity). Transportability claims must therefore be evaluated at intersectional levels.

### 11.6. Illustrative case studies for operational audits

The preceding risks can be made operational through empirical audit procedures grounded in documented failures of clinical algorithms. Shortcut learning can be audited similarly in imaging models. Zech et al. [17] showed that pneumonia classifiers trained on chest radiographs generalized inconsistently across hospital systems and that convolutional neural networks could identify the hospital system from radiographs with near-perfect accuracy. A practical audit would therefore freeze the encoder and train auxiliary classifiers to predict hospital, department, device, or acquisition protocol from latent embeddings. High environment decodability would indicate residual contextual contamination, particularly if aggregate AUROC remains acceptable.

Calibration drift and deployment failure provide another concrete audit target. Wong et al. [77] externally validated a widely implemented sepsis prediction model and reported modest discrimination, poor calibration, and substantial alert burden, with many sepsis cases missed at the commonly used threshold. Davis et al. [78] similarly showed that acute kidney injury prediction models could preserve discrimination while calibration progressively deteriorated over time. These findings support rolling-window audits reporting AUROC/AUPRC, calibration slope and intercept, Brier score, expected calibration error, alert burden, and net benefit. Invariance failure should be suspected when calibration or clinical utility deteriorates despite apparently stable discrimination.

Device-related subgroup bias also requires explicit evaluation. Sjoding et al. [79] showed that occult hypoxemia was more frequent among Black patients than White patients when pulse oximetry appeared normal. Although this is a measurement-device bias rather than an AI model failure itself, it illustrates how biased inputs can contaminate downstream prediction. An oxygenation-based model should therefore be audited by comparing pulse oximetry with arterial blood gases across subgroups and by reporting subgroup-specific calibration and error rates. Physiological invariance requires that models capture oxygenation physiology rather than device-specific measurement error.

These examples show that physiological invariance can be audited in concrete deployment settings, but they also expose unresolved methodological questions. In particular, the field still lacks agreed thresholds for constraint strength, transportability, subgroup reliability, and prospective recalibration. The next section therefore identifies the open problems that must be addressed before physiological invariance can become a reproducible design and evaluation principle.

## 12. Open questions and urgent research directions

While the concept of physiological invariance provides a unifying design principle, several open questions remain unresolved and define a concrete research agenda.

### 12.1. Formal definition of physiological invariance

Current invariant learning frameworks define stability in statistical or causal terms [11,12], yet medicine lacks a precise mathematical characterization of what constitutes a physiological invariant. Future work should formalize whether invariance should be defined at the level of conditional distributions, latent dynamical systems, conservation constraints, or intervention responses. Without such formalization, invariance risks remaining metaphorical rather than testable.

### 12.2. Quantifying the strength of mechanistic priors

How strong should mechanism-informed regularization be relative to data-driven objectives? Overly weak priors may fail to prevent shortcut learning [15], whereas overly strong constraints may suppress meaningful heterogeneity. Adaptive weighting strategies and cross-environment validation protocols should be used to balance bias and flexibility.

### 12.3. Adaptive balancing of mechanistic constraints and data-driven learning

Mechanism-informed learning should not rely on fixed constraint weights chosen a priori. A practical strategy is to treat physiological constraints as tunable regularization terms whose strength is adapted during training and re-evaluated during deployment. For example, the total objective can combine an empirical prediction loss, an invariance penalty across environments, and one or more mechanism-informed penalties, with constraint weights selected through nested cross-environment validation rather than in-domain performance alone. Candidate weights should be retained only if they improve target-environment calibration, worst-group performance, and biological plausibility without degrading discrimination or clinical utility.

Adaptive weighting methods from multi-task learning provide useful technical analogies. Uncertainty-based loss weighting can learn task-dependent weights instead of requiring manually fixed loss coefficients [80], whereas gradient normalization methods dynamically balance losses by equalizing training rates across objectives [81]. In physiological invariance, similar strategies could be used to prevent mechanistic penalties from dominating the learning signal when the prior is weak, mis-specified, or poorly identifiable. Constraint strength could also be updated using validation signals: if a mechanism-informed penalty improves biological plausibility but worsens subgroup calibration or external performance, its weight should be reduced; if an unconstrained model shows high environment decodability or implausible physiological trajectories, the constraint can be strengthened.

Prospective evaluation should follow a staged deployment pathway. Before clinical use, models should undergo silent-mode evaluation on temporally and geographically external data, reporting AUROC/AUPRC, calibration slope and intercept, Brier score, expected calibration error, net benefit, subgroup performance, and representation-level environment decodability. During early clinical implementation, DECIDE-AI can guide reporting of human–AI interaction, workflow integration, failure modes, and clinician override patterns [82]. For interventional evaluation, SPIRIT-AI and CONSORT-AI provide protocol and trial-reporting extensions for AI-based interventions [83,84]. For continuously updated systems, prospective monitoring should be specified in advance, including drift thresholds, recalibration rules, retraining triggers, version control, and impact assessment. This aligns with the FDA concept of predetermined change control plans for AI-enabled devices, which requires planned modifications, validation methods, and assessment of the impact of changes before implementation [77]. Thus, adaptive physiological invariance should be understood as a lifecycle process: constraints are not fixed once at development, but periodically stress-tested, recalibrated, weakened, strengthened, or retired according to prespecified evidence thresholds.

### 12.4. Reconciling invariance with biological diversity

Human physiology exhibits structured variability driven by sex, ancestry, age, comorbidity burden, and gene–environment interactions. Determining which aspects of physiology are truly transportable across populations, and which are population-specific, remains an empirical question. Future studies should explicitly test invariance across ancestrally and geographically diverse cohorts rather than assuming universality.

### 12.5. Multimodal alignment under missingness

Real-world healthcare data are incomplete and irregular. How invariant representations can be reliably learned when modalities are partially missing, asynchronously sampled, or institution-specific remains unclear. Advances in multimodal self-supervision [1,38] should be evaluated under realistic sparsity patterns.

### 12.6. Benchmarking and reproducibility

The field lacks standardized benchmarks explicitly designed to evaluate cross-environment invariance rather than within-site accuracy. Establishing open, versioned, cross-institutional datasets with predefined environments and longitudinal monitoring should be established to enable reproducible evaluation of transportability.

### 12.7. Lifecycle validation and regulatory science

AI systems evolve over time. Defining invariance thresholds should be compatible with FDA and IVDR lifecycle monitoring frameworks remains an open regulatory science problem. Prospective studies should determine what magnitude of calibration drift or subgroup disparity should trigger model retraining or re-certification.

### 12.8. Theoretical limits of transportability

Finally, it remains unclear whether certain clinical prediction tasks admit invariant representations at all. In domains where treatment effects, documentation practices, or population structure fundamentally differ, no stable representation may exist. Future work should characterize these limits is essential to avoid over-promising universal generalization.

## 13. Limitations and open challenges

The framework proposed here faces several important limitations. First, mechanistic knowledge in human physiology is incomplete, context-dependent, and continuously evolving. Many biological systems involve nonlinear interactions, latent confounding, and poorly characterized feedback loops. Embedding incorrect or oversimplified mechanistic assumptions into learning objectives may bias models rather than stabilize them.

Second, physiological mechanisms are shared across humans but are not uniform. Population-specific variability, including genetic diversity, environmental exposures, comorbidity patterns, and life-course effects, may alter the expression of otherwise stable mechanisms. A representation that is invariant at one scale may obscure meaningful heterogeneity at another. Universal representations should therefore accommodate structured variability rather than erase it.

Third, real-world healthcare data are subject to substantial measurement error, missingness, device variability, coding artifacts, and workflow-induced bias. Mechanism-informed regularization cannot compensate for systematically biased or low-quality inputs. In some settings, poor data quality may overwhelm physiological signal, limiting the feasibility of invariant learning.

These challenges suggest that physiological invariance should be viewed not as an established property of medical AI systems, but as a hypothesis to be tested empirically across diverse populations and deployment environments.

These limitations imply that physiological invariance should be deployed as an adaptive lifecycle framework rather than as a static modeling assumption. Mechanistic constraints should remain provisional, versioned, and empirically revisable, with prospective monitoring used to determine when recalibration, retraining, constraint weakening, or model withdrawal is required.

## Conclusion

This article has focused on representation learning as the primary locus of transportability in medical AI, while treating fairness, calibration, and lifecycle monitoring as deployment-level concerns that interact with, but are not subsumed by, the invariance framework. Invariants grounded in causality and mechanism may offer one path toward improved transportability, provided their empirical limits and domain-specific constraints are carefully characterized. The next generation of models should be judged not only by local accuracy, but by their ability to respect biology across settings.

## References

[1] Moor M, Banerjee O, Abad ZSH, Krumholz HM, Leskovec J, Topol EJ, et al. Foundation models for generalist medical artificial intelligence. Nature. 2023;616(7956):259–65. doi: 10.1038/s41586-023-05881-4 37045921

[2] Yan S, Yu Z, Primiero C, Vico-Alonso C, Wang Z, Yang L, et al. A multimodal vision foundation model for clinical dermatology. Nat Med. 2025;31(8):2691–702. doi: 10.1038/s41591-025-03747-y 40481209 PMC12353815

[3] Li Y, Wynne JF, Wu Y, Qiu RLJ, Tian S, Wang T, et al. Automatic medical imaging segmentation via self-supervising large-scale convolutional neural networks. Radiother Oncol. 2025;204:110711. doi: 10.1016/j.radonc.2025.110711 39798701 PMC11938206

[4] He Y, Huang F, Jiang X, Nie Y, Wang M, Wang J, et al. Foundation model for advancing healthcare: challenges, opportunities and future directions. IEEE Rev Biomed Eng. 2025;18:172–91. doi: 10.1109/RBME.2024.3496744 39531565

[5] Westerhof N, Lankhaar JW, Westerhof BE. The arterial Windkessel. Med Biol Eng Comput. 2009;47:131–41. doi: 10.1007/s11517-008-0359-2 18543011

[6] Khonsary SA. Guyton and Hall: textbook of medical physiology. Surg Neurol Int. 2017;8:275. doi: 10.4103/sni.sni_327_17

[7] Veldhuis JD, Keenan DM, Pincus SM. Motivations and methods for analyzing pulsatile hormone secretion. Endocr Rev. 2008;29(7):823–64. doi: 10.1210/er.2008-0005 18940916 PMC2647703

[8] Clarke IJ, Cummins JT. The temporal relationship between gonadotropin releasing hormone (GnRH) and luteinizing hormone (LH) secretion in ovariectomized ewes. Endocrinology. 1982;111(5):1737–9. doi: 10.1210/endo-111-5-1737 6751801

[9] McPherson R, Pincus M. Henry’s Clinical Diagnosis And Management By Laboratory Methods. 24th ed. Fac Bookshelf; 2021. https://hsrc.himmelfarb.gwu.edu/books/268

[10] Levey AS, Inker LA, Coresh J. GFR estimation: from physiology to public health. Am J Kidney Dis. 2014;63(5):820–34. doi: 10.1053/j.ajkd.2013.12.006 24485147 PMC4001724

[11] Peters J, Bühlmann P, Meinshausen N. Causal inference by using invariant prediction: identification and confidence intervals. J R Stat Soc Ser B Stat Methodol. 2016;78(5):947–1012. doi: 10.1111/rssb.12167

[12] Arjovsky M, Bottou L, Gulrajani I, Lopez-Paz D. Invariant Risk Minimization. arXiv; 2020. doi: 10.48550/arXiv.1907.02893

[13] Vallée A, Feki A, Moawad G, Ayoubi J-M. A semi-mechanistic mathematical framework for simulating multi-hormone dynamics in reproductive endocrinology. Comput Struct Biotechnol J. 2025;27:3654–62. doi: 10.1016/j.csbj.2025.08.013 40895285 PMC12395073

[14] Raissi M, Perdikaris P, Karniadakis GE. Physics-informed neural networks: a deep learning framework for solving forward and inverse problems involving nonlinear partial differential equations. J Comput Phys. 2019;378:686–707. doi: 10.1016/j.jcp.2018.10.045

[15] Ong Ly C, Unnikrishnan B, Tadic T, Patel T, Duhamel J, Kandel S, et al. Shortcut learning in medical AI hinders generalization: method for estimating AI model generalization without external data. NPJ Digit Med. 2024;7(1):124. doi: 10.1038/s41746-024-01118-4 38744921 PMC11094145

[16] Geirhos R, Jacobsen JH, Michaelis C, Zemel R, Brendel W, Bethge M. Shortcut learning in deep neural networks. Nat Mach Intell. 2020;2:665–73. doi: 10.1038/s42256-020-00257-z

[17] Zech JR, Badgeley MA, Liu M, Costa AB, Titano JJ, Oermann EK. Variable generalization performance of a deep learning model to detect pneumonia in chest radiographs: a cross-sectional study. PLoS Med. 2018;15(11):e1002683. doi: 10.1371/journal.pmed.1002683 30399157 PMC6219764

[18] Obermeyer Z, Powers B, Vogeli C, Mullainathan S. Dissecting racial bias in an algorithm used to manage the health of populations. Science. 2019;366(6464):447–53. doi: 10.1126/science.aax2342 31649194

[19] Yu AC, Mohajer B, Eng J. External validation of deep learning algorithms for radiologic diagnosis: a systematic review. Radiol Artif Intell. 2022;4(3):e210064. doi: 10.1148/ryai.210064 35652114 PMC9152694

[20] Rockenschaub P, Akay EM, Carlisle BG, Hilbert A, Wendland J, Meyer-Eschenbach F, et al. External validation of AI-based scoring systems in the ICU: a systematic review and meta-analysis. BMC Med Inform Decis Mak. 2025;25(1):5. doi: 10.1186/s12911-024-02830-7 39762808 PMC11702098

[21] Rockenschaub P, Hilbert A, Kossen T, Elbers P, von Dincklage F, Madai VI, et al. The impact of multi-institution datasets on the generalizability of machine learning prediction models in the ICU. Crit Care Med. 2024;52(11):1710–21. doi: 10.1097/CCM.0000000000006359 38958568 PMC11469625

[22] Sel K, Mohammadi A, Pettigrew RI, Jafari R. Physics-informed neural networks for modeling physiological time series for cuffless blood pressure estimation. NPJ Digit Med. 2023;6(1):110. doi: 10.1038/s41746-023-00853-4 37296218 PMC10256762

[23] Godau P, Kalinowski P, Christodoulou E, Reinke A, Tizabi M, Ferrer L, et al. Navigating prevalence shifts in image analysis algorithm deployment. Med Image Anal. 2025;102:103504. doi: 10.1016/j.media.2025.103504 40020420

[24] Abdalla M, Fine B. Hurdles to artificial intelligence deployment: noise in schemas and “gold” labels. Radiol Artif Intell. 2023;5:e220056. doi: 10.1148/ryai.220056 PMC10077093 37035427

[25] Choi Y, Yu W, Nagarajan MB, Teng P, Goldin JG, Raman SS, et al. Translating AI to clinical practice: overcoming data shift with explainability. Radiographics. 2023;43(5):e220105. doi: 10.1148/rg.220105 37104124 PMC10190133

[26] Vaghela AK. Physics-informed neural network-based pulsatile flow modeling and targeted drug delivery optimization in computational hemodynamics. J Pharm Bioallied Sci. 2025;17(Suppl 4):S3404–6. doi: 10.4103/jpbs.jpbs_1424_25 41522916 PMC12788563

[27] Albers DJ, Levine ME, Stuart A, Mamykina L, Gluckman B, Hripcsak G. Mechanistic machine learning: how data assimilation leverages physiologic knowledge using Bayesian inference to forecast the future, infer the present, and phenotype. J Am Med Inform Assoc. 2018;25(10):1392–401. doi: 10.1093/jamia/ocy106 30312445 PMC6188514

[28] Roschewitz M, Khara G, Yearsley J, Sharma N, James JJ, Ambrózay É, et al. Automatic correction of performance drift under acquisition shift in medical image classification. Nat Commun. 2023;14(1):6608. doi: 10.1038/s41467-023-42396-y 37857643 PMC10587231

[29] Lakatta EG. Arterial and cardiac aging: major shareholders in cardiovascular disease enterprises: Part III: cellular and molecular clues to heart and arterial aging. Circulation. 2003;107(3):490–7. doi: 10.1161/01.cir.0000048894.99865.02 12551876

[30] Hunter DJ. Gene-environment interactions in human diseases. Nat Rev Genet. 2005;6(4):287–98. doi: 10.1038/nrg1578 15803198

[31] Pearl J. Causality: models, reasoning, and inference.

[32] Schölkopf B, Locatello F, Bauer S, Ke NR, Kalchbrenner N, Goyal A, et al. Towards Causal Representation Learning. arXiv; 2021. doi: 10.48550/arXiv.2102.11107

[33] Rosenfeld E, Ravikumar P, Risteski A. The Risks of Invariant Risk Minimization. arXiv; 2021. doi: 10.48550/arXiv.2010.05761

[34] Wang M, Deng W. Deep visual domain adaptation: a survey. Neurocomputing. 2018;312:135–53. doi: 10.1016/j.neucom.2018.05.083

[35] Ganin Y, Lempitsky V. Unsupervised Domain Adaptation by Backpropagation. arXiv; 2015. doi: 10.48550/arXiv.1409.7495

[36] Pan SJ, Yang Q. A survey on transfer learning. IEEE Trans Knowl Data Eng. 2010;22(10):1345–59. doi: 10.1109/tkde.2009.191

[37] Subbaswamy A, Saria S. From development to deployment: dataset shift, causality, and shift-stable models in health AI. Biostatistics. 2020;21(2):345–52. doi: 10.1093/biostatistics/kxz041 31742354

[38] Krishnan R, Rajpurkar P, Topol EJ. Self-supervised learning in medicine and healthcare. Nat Biomed Eng. 2022;6(12):1346–52. doi: 10.1038/s41551-022-00914-1 35953649

[39] Azizi S, Mustafa B, Ryan F, Beaver Z, Freyberg J, Deaton J, et al. Big Self-supervised Models Advance Medical Image Classification. arXiv; 2021. doi: 10.48550/arXiv.2101.05224

[40] Huang SC, Jensen M, Yeung-Levy S, Lungren MP, Poon H, Chaudhari AS. A systematic review and implementation guidelines of multimodal foundation models in medical imaging. Res Sq. 2025. doi: 10.21203/rs.3.rs-5537908/v1

[41] Li Y, Rao S, Solares JRA, Hassaine A, Ramakrishnan R, Canoy D, et al. BEHRT: transformer for electronic health records. Sci Rep. 2020;10: 7155. doi: 10.1038/s41598-020-62922-y 32346050 PMC7189231

[42] Alsentzer E, Murphy JR, Boag W, Weng W-H, Jin D, Naumann T, et al. Publicly available clinical BERT embeddings. arXiv; 2019. doi: 10.48550/arXiv.1904.03323

[43] Peters J, Janzing D, Schölkopf B. Elements of causal inference: Foundations and learning algorithms. Cambridge, MA, USA: MIT Press; 2017. https://mitpress.mit.edu/9780262037310/elements-of-causal-inference/

[44] Locatello F, Bauer S, Lucic M, Raetsch G, Gelly S, Schölkopf B, et al. Challenging Common Assumptions in the Unsupervised Learning of Disentangled Representations. Proceedings of the 36th International Conference on Machine Learning. PMLR; 2019. pp. 4114–24. https://proceedings.mlr.press/v97/locatello19a.html

[45] Hodgkin AL, Huxley AF. A quantitative description of membrane current and its application to conduction and excitation in nerve. J Physiol. 1952;117(4):500–44. doi: 10.1113/jphysiol.1952.sp004764 12991237 PMC1392413

[46] Guyton AC, Coleman TG, Granger HJ. Circulation: overall regulation. Annu Rev Physiol. 1972;34:13–46. doi: 10.1146/annurev.ph.34.030172.000305 4334846

[47] Rowland M, Benet LZ, Graham GG. Clearance concepts in pharmacokinetics. J Pharmacokinet Biopharm. 1973;1(2):123–36. doi: 10.1007/BF01059626 4764426

[48] Sheiner LB, Beal S, Rosenberg B, Marathe VV. Forecasting individual pharmacokinetics. Clin Pharmacol Ther. 1979;26(3):294–305. doi: 10.1002/cpt1979263294 466923

[49] Bergman RN, Ider YZ, Bowden CR, Cobelli C. Quantitative estimation of insulin sensitivity. Am J Physiol. 1979;236(6):E667–77. doi: 10.1152/ajpendo.1979.236.6.E667 443421

[50] Savageau MA. Biochemical systems analysis. I. Some mathematical properties of the rate law for the component enzymatic reactions. J Theor Biol. 1969;25(3):365–9. doi: 10.1016/s0022-5193(69)80026-3 5387046

[51] Guo C, Pleiss G, Sun Y, Weinberger KQ. On Calibration of Modern Neural Networks. arXiv; 2017. doi: 10.48550/arXiv.1706.04599

[52] Roberts M, Driggs D, Thorpe M, Gilbey J, Yeung M, Ursprung S. Common pitfalls and recommendations for using machine learning to detect and prognosticate for COVID-19 using chest radiographs and CT scans. Nat Mach Intell. 2021;3:199–217. doi: 10.1038/s42256-021-00307-0

[53] Kelly CJ, Karthikesalingam A, Suleyman M, Corrado G, King D. Key challenges for delivering clinical impact with artificial intelligence. BMC Med. 2019;17(1):195. doi: 10.1186/s12916-019-1426-2 31665002 PMC6821018

[54] Martin AR, Kanai M, Kamatani Y, Okada Y, Neale BM, Daly MJ. Clinical use of current polygenic risk scores may exacerbate health disparities. Nat Genet. 2019;51(4):584–91. doi: 10.1038/s41588-019-0379-x 30926966 PMC6563838

[55] Rackauckas C, Ma Y, Martensen J, Warner C, Zubov K, Supekar R, et al. Universal Differential Equations for Scientific Machine Learning. In: arXiv.org [Internet]. 2020 [cited 26 May 2026]. Available: https://arxiv.org/abs/2001.04385v4

[56] Karniadakis GE, Kevrekidis IG, Lu L, Perdikaris P, Wang S, Yang L. Physics-informed machine learning. Nat Rev Phys. 2021;3:422–40. doi: 10.1038/s42254-021-00314-5

[57] Rubanova Y, Chen RTQ, Duvenaud D. Latent ODEs for Irregularly-Sampled Time Series. arXiv; 2019. doi: 10.48550/arXiv.1907.03907

[58] Kingma DP, Welling M. Auto-encoding variational Bayes. In: arXiv.org [Internet]. 2013 [cited 26 May 2026]. Available: https://arxiv.org/abs/1312.6114v11

[59] Chen RTQ, Rubanova Y, Bettencourt J, Duvenaud D. Neural ordinary differential equations.

[60] Johnson AEW, Bulgarelli L, Shen L, Gayles A, Shammout A, Horng S, et al. MIMIC-IV, a freely accessible electronic health record dataset. Sci Data. 2023;10(1):1. doi: 10.1038/s41597-022-01899-x 36596836 PMC9810617

[61] Pollard TJ, Johnson AEW, Raffa JD, Celi LA, Mark RG, Badawi O. The eICU collaborative research database, a freely available multi-center database for critical care research. Sci Data. 2018;5:180178. doi: 10.1038/sdata.2018.178 30204154 PMC6132188

[62] Johnson AEW, Pollard TJ, Berkowitz SJ, Greenbaum NR, Lungren MP, Deng C-Y, et al. MIMIC-CXR, a de-identified publicly available database of chest radiographs with free-text reports. Sci Data. 2019;6(1):317. doi: 10.1038/s41597-019-0322-0 31831740 PMC6908718

[63] Hayat N, Geras KJ, Shamout FE. MedFuse: Multi-modal fusion with clinical time-series data and chest X-ray images. arXiv; 2023. doi: 10.48550/arXiv.2207.07027

[64] Murphy AH. A new vector partition of the probability score. J Appl Meteorol Climatol. 1973;12:595–600. doi: 10.1175/1520-0450(1973)012<0595:ANVPOT>2.0.CO;2

[65] Vazquez J, Facelli JC. Conformal prediction in clinical medical sciences. J Healthc Inform Res. 2022;6:241–52. doi: 10.1007/s41666-021-00113-8 35898853 PMC9309105

[66] Angelopoulos AN, Bates S. A gentle introduction to conformal prediction and distribution-free uncertainty quantification. arXiv; 2022. doi: 10.48550/arXiv.2107.07511

[67] Che Z, Purushotham S, Cho K, Sontag D, Liu Y. Recurrent neural networks for multivariate time series with missing values. Sci Rep. 2018;8(1):6085. doi: 10.1038/s41598-018-24271-9 29666385 PMC5904216

[68] Shukla SN, Marlin BM. Multi-time attention networks for irregularly sampled time series. Int Conf Learn Represent. 2021;2021:14897–911. 41737905 PMC12927590

[69] Yèche H, Kuznetsova R, Zimmermann M, Hüser M, Lyu X, Faltys M, et al. HiRID-ICU-benchmark -- a comprehensive machine learning benchmark on high-resolution ICU data. arXiv; 2022. doi: 10.48550/arXiv.2111.08536

[70] Li X, Gu Y, Dvornek N, Staib LH, Ventola P, Duncan JS. Multi-site fMRI analysis using privacy-preserving federated learning and domain adaptation: ABIDE results. Med Image Anal. 2020;65:101765. doi: 10.1016/j.media.2020.101765 32679533 PMC7569477

[71] Rieke N, Hancox J, Li W, Milletarì F, Roth HR, Albarqouni S, et al. The future of digital health with federated learning. NPJ Digit Med. 2020;3:119. doi: 10.1038/s41746-020-00323-1 33015372 PMC7490367

[72] Hardt M, Price E, Srebro N. Equality of opportunity in supervised learning. Advances in Neural Information Processing Systems. Curran Associates, Inc.; 2016. https://papers.nips.cc/paper_files/paper/2016/hash/6a9659feb1216f14f7384ba499518b38-Abstract.html

[73] Kusner MJ, Loftus JR, Russell C, Silva R. Counterfactual Fairness. arXiv; 2018. doi: 10.48550/arXiv.1703.06856

[74] Pleiss G, Raghavan M, Wu F, Kleinberg J, Weinberger KQ. On fairness and calibration. arXiv; 2017. doi: 10.48550/arXiv.1709.02012

[75] Benjamens S, Dhunnoo P, Meskó B. The state of artificial intelligence-based FDA-approved medical devices and algorithms: an online database. NPJ Digit Med. 2020;3:118. doi: 10.1038/s41746-020-00324-0 32984550 PMC7486909

[76] Kleinberg J, Mullainathan S, Raghavan M. Inherent trade-offs in the fair determination of risk scores. arXiv; 2016. doi: 10.48550/arXiv.1609.05807

[77] Wong A, Otles E, Donnelly JP, Krumm A, McCullough J, DeTroyer-Cooley O, et al. External validation of a widely implemented proprietary sepsis prediction model in hospitalized patients. JAMA Intern Med. 2021;181(8):1065–70. doi: 10.1001/jamainternmed.2021.2626 34152373 PMC8218233

[78] Davis SE, Lasko TA, Chen G, Siew ED, Matheny ME. Calibration drift in regression and machine learning models for acute kidney injury. J Am Med Inform Assoc. 2017;24(6):1052–61. doi: 10.1093/jamia/ocx030 28379439 PMC6080675

[79] Sjoding MW, Dickson RP, Iwashyna TJ, Gay SE, Valley TS. Racial bias in pulse oximetry measurement. N Engl J Med. 2020;383(25):2477–8. doi: 10.1056/NEJMc2029240 33326721 PMC7808260

[80] Kendall A, Gal Y, Cipolla R. Multi-task learning using uncertainty to weigh losses for scene geometry and semantics. arXiv; 2018. doi: 10.48550/arXiv.1705.07115

[81] Chen Z, Badrinarayanan V, Lee C-Y, Rabinovich A. GradNorm: gradient normalization for adaptive loss balancing in deep multitask networks. In: arXiv.org [Internet]. 2017 [cited 26 May 2026]. Available from: https://arxiv.org/abs/1711.02257v4

[82] Vasey B, Nagendran M, Campbell B, Clifton DA, Collins GS, Denaxas S, et al. Reporting guideline for the early stage clinical evaluation of decision support systems driven by artificial intelligence: DECIDE-AI. BMJ. 2022;377:e070904. doi: 10.1136/bmj-2022-070904 35584845 PMC9116198

[83] Cruz Rivera S, Liu X, Chan A-W, Denniston AK, Calvert MJ, SPIRIT-AI and CONSORT-AI Working Group. Guidelines for clinical trial protocols for interventions involving artificial intelligence: the SPIRIT-AI extension. Nat Med. 2020;26:1351–63. doi: 10.1038/s41591-020-1037-7 32908284 PMC7598944

[84] Liu X, Cruz Rivera S, Moher D, Calvert MJ, Denniston AK, SPIRIT-AI and CONSORT-AI Working Group. Reporting guidelines for clinical trial reports for interventions involving artificial intelligence: the CONSORT-AI extension. Nat Med. 2020;26:1364–74. doi: 10.1038/s41591-020-1034-x 32908283 PMC7598943

